# Supplementary material for: Genome-Wide Association Analysis of Muscle pH in Texel Sheep × Altay Sheep F2 Resource Population
Source: Animals (Basel). 2023 Jun 30;13(13):2162. doi: 10.3390/ani13132162 (PMC10339987; doi:10.3390/ani13132162)
Supplement: Supplementary file 1 [file animals-13-02162-s001.zip › Table S1 Distribution and average spacing of SNP loci on each chromosome after quality control.pdf]

Table S1 Distribution and average spacing of SNP loci on each chromosome after quality control

Chromosome	Length of chromosome(bp)	no.SNPs	Average no.SNPs(Mb)[a]
1	275612895	69951	253.8
2	248993846	61630	247.52
3	224283230	56467	251.77
4	119255633	30015	251.69
5	107901688	26974	249.99
6	117031472	28668	244.96
7	100079507	24993	249.73
8	90695168	22724	250.55
9	94726778	23155	244.44
10	86447213	20670	239.11
11	62248096	16015	257.28
12	79100223	19920	251.83
13	83079144	20792	250.27
14	62722625	16117	256.96
15	80923592	20417	252.3
16	71719816	17789	248.03
17	72286588	17566	243
18	68604602	16850	245.61
19	60464314	15537	256.96
20	51176841	12552	245.27
21	50073674	12249	244.62
22	50832532	12762	251.06
23	62330649	15149	243.04
24	42034648	10700	254.55
25	45367442	11549	254.57
26	44077779	11030	250.24
27[b]	135437088	937	6.92

Note: a.Average distance: refers to the average distance between adjacent SNP loci; b. Chromosome 27: X chromosome.
