# Supplementary figures and images for: Genome-Wide Association Analysis of Muscle pH in Texel Sheep × Altay Sheep F2 Resource Population
Source: Animals (Basel). 2023 Jun 30;13(13):2162. doi: 10.3390/ani13132162 (PMC10339987; doi:10.3390/ani13132162)

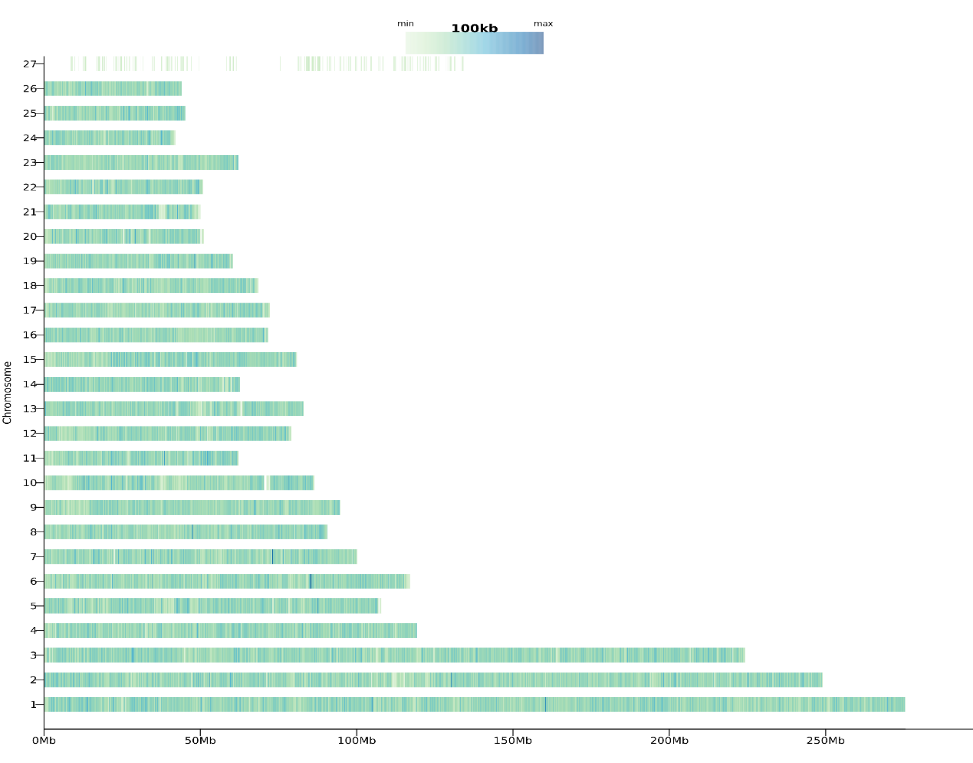

Supplement: Supplementary file 1 [file animals-13-02162-s001.zip › Figure S1 SNP distribution density map after quality control.png]
